# Reduced Microvascular Density in Omental Biopsies of Children with Chronic Kidney Disease

**DOI:** 10.1371/journal.pone.0166050

**Published:** 2016-11-15

**Authors:** Dorothea Burkhardt, Maria Bartosova, Betti Schaefer, Niels Grabe, Bernd Lahrmann, Hamoud Nasser, Christian Freise, Axel Schneider, Anja Lingnau, Petra Degenhardt, Bruno Ranchin, Peter Sallay, Rimante Cerkauskiene, Michal Malina, Gema Ariceta, Claus Peter Schmitt, Uwe Querfeld

**Affiliations:** 1 Department of Pediatric Nephrology, Charité-Universitätsmedizin Berlin, Berlin, Germany; 2 Center for Pediatric and Adolescent Medicine, University of Heidelberg, Heidelberg, Germany; 3 Bioquant, Hamamatsu Tissue Imaging and Analysis (TIGA) Center, University of Heidelberg, Heidelberg, Germany; 4 Center for Cardiovascular Research, Charité Universitätsmedizin Berlin, Berlin, Germany; 5 Department of Pediatric Surgery, Charité-Universitätsmedizin Berlin, Berlin, Germany; 6 Department of Pediatric Urology, Charité-Universitätsmedizin Berlin, Berlin, Germany; 7 Department of Pediatric Surgery, Klinikum Ernst von Bergmann, Potsdam, Germany; 8 Hospices Civils de Lyon, Service de Nephrologie Pediatrique and Epicime-Centre d’Investigation Clinique 1407, Hopital Femme Mere Enfant, Lyon, France; 9 First Department of Pediatrics, Semmelweis University, Budapest, Hungary; 10 Coordinating Centre for Children’s Rare Diseases, Children´s Hospital, Affiliate of Vilnius University Hospital Santariskiu Klinikos, Vilnius, Lithuania; 11 Department of Pediatrics, Second Faculty of Medicine, Charles University-Prague, Prague 5, Czech Republic; 12 Servicio de Nefrología Pediátrica, Hospital Universitari Vall d’Hebron, Barcelona, Spain; University Medical Center Utrecht, NETHERLANDS

## Abstract

**Background:**

Endothelial dysfunction is an early manifestation of cardiovascular disease (CVD) and consistently observed in patients with chronic kidney disease (CKD). We hypothesized that CKD is associated with systemic damage to the microcirculation, preceding macrovascular pathology. To assess the degree of “uremic microangiopathy”, we have measured microvascular density in biopsies of the omentum of children with CKD.

**Patients and Methods:**

Omental tissue was collected from 32 healthy children (0–18 years) undergoing elective abdominal surgery and from 23 age-matched cases with stage 5 CKD at the time of catheter insertion for initiation of peritoneal dialysis. Biopsies were analyzed by independent observers using either a manual or an automated imaging system for the assessment of microvascular density. Quantitative immunohistochemistry was performed for markers of autophagy and apoptosis, and for the abundance of the angiogenesis-regulating proteins VEGF-A, VEGF-R2, Angpt1 and Angpt2.

**Results:**

Microvascular density was significantly reduced in uremic children compared to healthy controls, both by manual imaging with a digital microscope (median surface area 0.61% vs. 0.95%, p<0.0021 and by automated quantification (total microvascular surface area 0.89% vs. 1.17% p = 0.01). Density measured by manual imaging was significantly associated with age, height, weight and body surface area in CKD patients and healthy controls. In multivariate analysis, age and serum creatinine level were the only independent, significant predictors of microvascular density (r^2^ = 0.73). There was no immunohistochemical evidence for apoptosis or autophagy. Quantitative staining showed similar expression levels of the angiogenesis regulators VEGF-A, VEGF-receptor 2 and Angpt1 (p = 0.11), but Angpt2 was significantly lower in CKD children (p = 0.01).

**Conclusions:**

Microvascular density is profoundly reduced in omental biopsies of children with stage 5 CKD and associated with diminished Angpt2 signaling. Microvascular rarefaction could be an early systemic manifestation of CKD-induced cardiovascular disease.

## Introduction

Chronic kidney disease (CKD), even at early stages, is associated with a high cardiovascular morbidity and mortality [1, 2]. At present, mechanisms explaining the link between renal and cardiovascular disease (CVD) are incompletely understood.

Endothelial dysfunction is a clinical hallmark of CVD and has been observed consistently in patients with CKD [3]. Endothelial dysfunction is a systemic disorder involving conduit arteries, peripheral resistance vessels and capillary beds, and predisposing to cardiovascular complications [4, 5]. A multitude of risk factors for CVD converge in promoting endothelial dysfunction, thus creating “the ultimate risk of the risk factors” [4]. While there is compelling evidence for a disturbed endothelial function in CKD (e.g. impaired endothelium-dependent vasodilation, pro-inflammatory activation), few studies have analyzed the morphology of the microcirculation, which provides the structural basis of endothelial function. Capillary rarefaction was documented in animals with experimental CKD in the myocardium [6, 7] and in the skeletal muscle [8]. Studies of the peripheral microcirculation in patients with CKD stage 3–5 using nailfold microscopy have likewise shown reduced capillary density [9, 10]. In a postmortem study, myocardial capillary density was reduced by almost 50% in dialyzed patients compared to normotensive non-CKD controls [11]. In addition, capillary rarefaction in the kidney is a typical feature of chronic progressive renal disease [12] and of chronic kidney allograft failure [13], and all studies of the retinal microcirculation of patients with CKD found evidence for a microvascular retinopathy [14–17].

We hypothesized that CKD is associated with systemic damage to the microcirculation, preceding macrovascular pathology. To assess the degree of “uremic microangiopathy”, we have measured microvascular density in biopsies of the omentum, a tissue with high metabolic activity and an important source of angiogenic factors [18, 19], which is easily accessible during abdominal operations. We chose to study a pediatric CKD cohort, without confounding factors such as advanced age or additional comorbid conditions potentially affecting the microcirculation.

## Materials and Methods

### Patients

The International Pediatric Peritoneal Dialysis Study was approved by the Ethics Committee of the University of Heidelberg (S-487/2010) and Ethics Committees of all participating centers and all parts of the study were conducted in compliance with the Declaration of Helsinki. Written informed consent was obtained from parents and from patients as appropriate. First, a healthy control group of children with normal renal function was carefully selected from patients undergoing elective abdominal operations who were free of acute or chronic inflammatory diseases. Children with diminished renal function, malignancies or previous chemotherapy or corticosteroid treatment were excluded. Omental biopsies were performed in 32 control children (14 male, 18 female); diagnoses and abdominal operations performed in the control group are listed in the S1 and S2 Tables. All children were normotensive. Clinical and anthropometric data of controls was recorded at the time of surgery.

In a case-control study, we identified 24 cases (by age matching with healthy controls) with stage 5 CKD from the biobank of the International Pediatric Peritoneal Dialysis Study (www.clinicaltrials.gov–NCT01893710) with stored omental tissue taken at the time of first PD catheter insertion. Omental biopsies were performed in children initiating PD as part of an ongoing study evaluating morphological characteristics of the peritoneal membrane and the omentum [20]. Data of CKD patients had been recorded at the time of catheter insertion for PD by participating centers and was retrieved from the online database. The GFR was calculated by the Schwartz formula [21]. One patient was excluded from the study after review of the medical history because of previous cytotoxic therapy. All children were normotensive at the time of catheter insertion, 4 CKD patients were treated with antihypertensive drugs. The diagnoses of renal diseases of CKD patients are listed in the S3 Table.

### Quantification of microvascular density

Omental tissue was excised and immediately cut up into 3 pieces (approximately 1 x1 cm) and placed into vials containing 4% buffered formalin or RNAlater; the remaining tissue was immediately deep frozen in liquid nitrogen. Formalin-fixed samples were later embedded in paraffin, and deep frozen samples stored at -80°C. For the assessment of microvascular density, paraffin-fixed biopsies were stained with CD-31 antibody and measured by two different methods by independent observers.

First, the “manual imaging method” was performed by one trained observer (D.B.) with a digital microscope (Keyence BZ-9000) using the Analyzer Software®-II (Keyence) (n = 55, 23 CKD, 32 controls). Vessels with the morphological characteristics of arterioles or venules were excluded from the analysis. After demarcation (up to 9 boundaries) of tissue borders and correction for brightness, thinness and optical contrast, microphotographs were taken (1/350 sec., 4-fold magnification). Up to 1200 images were then used for the final high-resolution image of each tissue section. Imaging was performed for 3 tissue sections from each participant (total n = 165 sections) and the arithmetic mean microvessel count of each participant’s tissue was used for further calculations. For the assessment of interobserver variability, a random sample of 10% of tissue sections was evaluated by a second observer (H.N.) in a blinded fashion [22].

Second, 30 biopsies (17 CKD patients 13 controls) including patients and controls of all age groups were also measured with the fully automated Nanozoomer Digital Pathology System and NDP Viewer Software (Hamamatsu Photonics, Japan) by two trained observers (B.S., M.B.); the other samples were technically unsuitable for automated analysis. Automated quantitative analyses were performed using the Aperio Image Analysis Software (Aperio® Technologies, Inc., Vista, California, USA) and viewed by Image Scope version 11 (v11.2.0.780). Immunohistochemical stainings were evaluated using the Aperio Positive Pixel Count Algorithm (version 9) for quantification of the amount of positive pixels per scanned virtual slide (S1 and S2 Figs). Intensity ranges for weak, medium and strong signals and negative pixels were validated for each specific staining. Positivity was calculated as total number of positive pixels divided by total number of pixels (S1 Text).

### Immunohistochemistry

Staining with hematoxylin and eosin (HE) and acid fuchsin orange G (AFOG) was performed according to standard protocols. Immunohistochemistry was performed on formalin fixed tissue sections according to standard methods. Dewaxed and rehydrated tissue sections were incubated in 3% hydrogen peroxide to block endogenous peroxidases. The heat-induced antigen retrieval was performed in pressure cooker, using the Dako REAL Target Retrieval Buffer (Dako Cytomation, Glostrup, Denmark). Monoclonal antibodies against cluster of differentiation CD31 (1:25), podoplanin D2-40 (1:50) were purchased from Dako Cytomation (Glostrup, Denmark). Polyclonal antibodies against VEGF-A (1:1000) from Abcam (Cambridge, UK), Angp1 (1:50) from Sigma Aldrich (St. Louis, Missouri, USA) and Angpt2 (1:200) from Pierce Antibodies (Thermo Fisher, Rockford, USA) were applied for 1 hour at room temperature. Incubation with biotinylated secondary reagents (Vector Laboratories, Burlingame, CA, USA) for 30 min was followed by the ABC reagent (Vector). 3′3′Diaminobenzidine (DAB) was used for detection. Antibody diluent without primary antibody was used for negative control. Cell nuclei were counterstained with HE. In addition, paraffin embedded tissue specimens of biopsies were investigated by immunohistochemistry with antibodies against the autophagy marker LC3A/B (LC3A/B (D3U4C) XP® Rabbit mAb; Cell Signaling, Beverly, MA, USA), the apoptosis marker caspase-3 (Cleaved Caspase-3 (Asp175), Cell Signaling) and the intra- and extracellular domains of VEGFR2 (Rabbit anti-VEGFR2 (Flk-1,KDR), Zytomed Systems, Berlin, Germany) and anti-KDR/VEGFR2 antibody (Extracellular Domain LS-C122532; LifeSpan BioSciences, Seattle, WA, USA). Bound antibodies were visualized with the detection kits “Vector®NovaRED™” (Vector Laboratories), “Liquid Permanent Red” (Dako) and the „New Fuchsin Kit”(ScyTek Laboratories, Logan, UT, USA), respectively. Cell nuclei were counterstained with hematoxylin and eosin and antibody diluents without primary antibodies were used as respective negative controls.

### Statistical Methods

The GraphPad PRISM^®^^tm^ (Version 6.0) software and IBM^®^ SPSS^®^ Statistics for Windows (Version 23.0) software were used for statistical analysis. Intra- and interobserver variability was estimated by Bland-Altman statistics. The significance of differences between patients and controls was estimated by the non-parametric Mann-Whitney test. Pearson’s correlation coefficient of log-transformed data and stepwise multivariate linear regression with backward selection was used to estimate statistical significance of correlations between variables. Results of all measurements were expressed as mean, median and SD.

## Results

Patients and controls were comparable (Table 1) in age, weight, height, body mass index (BMI), age, adjusted BMI (BMI-SDS) and body surface area (BSA).

**Table 1:** Patients and Controls.

	Total	Controls	CKD	p*
**N**	55	32	23	
**Gender**				
male	30 (55%)	14	16	
female	25 (45%)	18	7	p = 0.01
**Age (years)**	n = 9	n = 3	n = 6	
0–2	19 (35%)	13	6	
3–6	11 (20%)	6	5	
7–12	15 (27%)	8	7	
13–18	10 (18%)	5	5	
**mean**	6.7 (±5.7)	6.2 (±5.7)	7.3 (±5.7)	p = 0.5
**Weight (kg)**	22.4 (±18.6)	22.5 (±20.8)	22 (±14.4)	p = 0.66
**Height (cm)**	104.9 (±35.8)	102.5 (±37.2)	108.6 (±34.4)	p = 0.53
**BMI**	17.3 (±4)	17.6 (±5.1)	17 (±1.9)	p = 0.82
**BMI-SDS**	- 0.28 (±1,4)	- 0.27 (±1.7)	- 0.27 (±1.1)	p = 0.93
**BSA (m**^**2**^**)**	0.78 (±0.5)	0.77 (±0.5)	0.8 (±0.4)	p = 0.55

*p = statistical significance (Mann-Whitney test) of the difference between controls and patients with CKD.

### Microvascular Density

**Fig 1:**
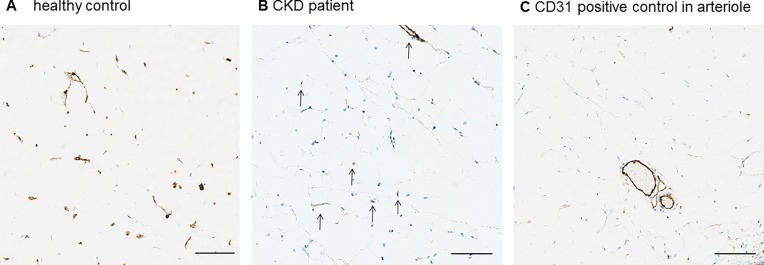
Capillary staining in omental tissue. Representative sample of omental tissue of a control child (**A**) and a child with CKD 5 (**B**). A positive control for the CD31 stain (**C**) is shown in a larger arteriole, venule and lymphatic vessel. Scale bars: 100μm.

**Fig 2:**
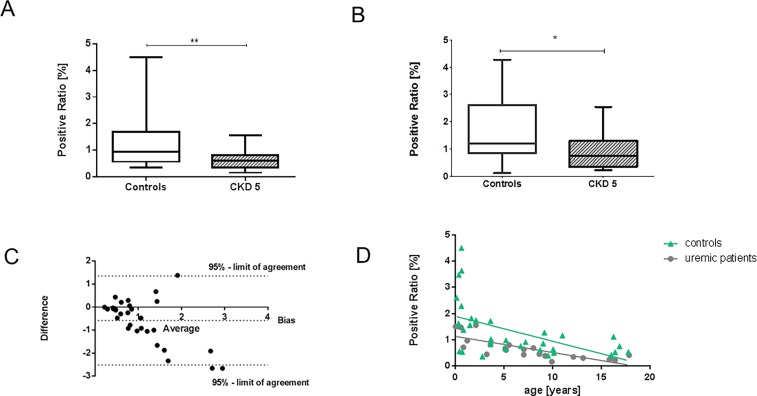
Microvascular density of omental tissue from children with CKD 5 and controls. Box plots of manual (**A**) and (**B**) automatic imaging measurements showing significantly decreased microvascular density in CKD patients. Positive ratio [%] denotes the percentage amount of tissue area staining positive with the CD31 marker. * p< 0.05 **p<0.01, Mann-Whitney test. (**C**) Bland-Altman graph showing degrees of agreement of measurements by both methods. (**D**) Linear regression analysis of microvascular density and age for patients with CKD (r = -0.83, p = <0.0001) and controls (r = -0.56; p = 0.001).

Microvascular density was significantly reduced in uremic children compared to healthy controls (Figs 1 and 2). Median capillary surface area, assessed manually with a digital microscope was 36% lower in CKD 5 (0.61% vs. 0.95% of total surface area in controls, p = 0.0021; Fig 2A). When four patients with antihypertensive treatment were excluded from the analysis, median microvascular density was reduced by 52% (p = 0.0007). The median intraobserver variability of the manual method was 12.2% and the median interobserver variability was 9.7%. The Bland-Altman statistics showed a good agreement between the observers, with a bias of 0.009 + 0.096 and 0.031 ± 0.147, respectively, and all except one observation in between limits of agreement. By automated imaging, the median capillary surface area was reduced by 51% (0.75% vs. 1.53%; p = 0.0216). (Fig 2B). Microvascular density in identical samples was significantly different when the manual and automated method were compared (median 0.6517 vs. 1.135; p = 0.02). The Bland-Altman statistics for all measurements with the manual vs. the automated method showed good agreement for biopsies with a low microvascular density, but there was increased variability of measurements with an increased number of microvessels (bias 0.5820 ± 0.9862) (Fig 2C). There was very low expression of the marker for lymphatic vessels, D2-40, in omental biopsies without a difference between patients and controls (p = 0.47). Therefore, measurements of microvascular density were unaffected by the very low lymphatic vessel supply of the omentum.

### Associations

Microvascular density measured by the manual method showed significant correlations with several anthropometric and clinical parameters as estimated by univariate linear regression. Thus, it was inversely associated with age in CKD children (r = -0.83, p = 0.0001) and controls (r = -0.56, p = 0.001; Fig 2D1) and with body surface area in CKD children (r = -0.75, p = 0.0006) and controls (r = -0.58, p = 0.0018). Microvascular density was inversely correlated with body weight (CKD: = -0.72, p = 0.001; controls = -0.53, p = 0.0022), and height (CKD: r = -0.76, p = 0.0004; controls; r = -0.63, p = 0.0006). There was no significant correlation in patients or controls with the age-adjusted body mass index (BMI-SDS), and the systolic or diastolic blood pressure. The duration of CKD, recorded in 12 of the 23 children with CKD, was significantly correlated with microvascular density (r = 0.71, p = 0.02). Microvessel density was furthermore inversely associated with serum levels of creatinine (r = -0.63; p = 0.0117). There were no significant correlations of manual measurements of microvascular density with the estimated glomerular filtration rate (eGFR) or the serum levels of urea, phosphorus, calcium, parathyroid hormone, bicarbonate, albumin, C-reactive protein or the white blood count.

In a multivariate linear regression analysis, significant predictors of microvascular density (manual measurements) for all children (both groups) were age and the serum creatinine level (Table 2). In a statistical model incorporating variables with significant univariate correlations, the variables age and serum creatinine explained 73% of the variability of the dependent variable, i.e. microvascular density.

**Table 2:** Multiple linear regression analysis: predictors of microvascular density.

Variable	B	Standard error	ß	p	R2 for the model
Age	-0.033	0.006	-0.581	0.000	0.729
Serum creatinine	-0.087	0.015	-0.571	0.000	

Automated measurements of vascular density were significantly associated with age (r = -0.38; p = 0.036), but not with other clinical or laboratory parameters.

### Mechanisms

**Fig 3:**
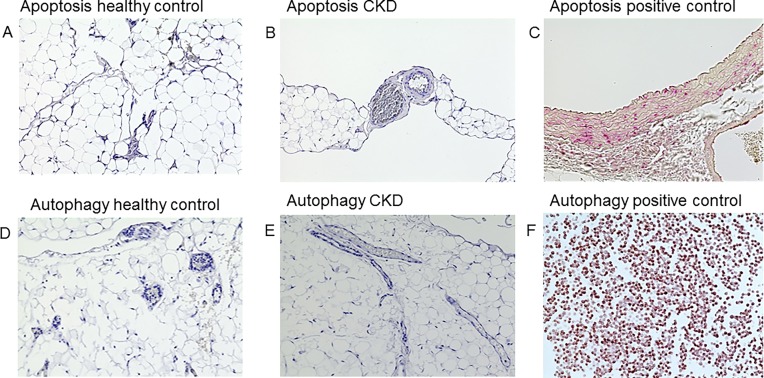
Apoptosis and autophagy in omental biopsies of children with CKD 5 and controls. Caspase-3 staining reveals no apoptotic signal in either controls (**A**) or children with CKD (**B**); (**C**) positive control. Similarly, staining with LC3A/B antibody reveals no autophagy signal in either controls (**D**) or children with CKD (**E**); (**F**) positive control.

**Fig 4:**
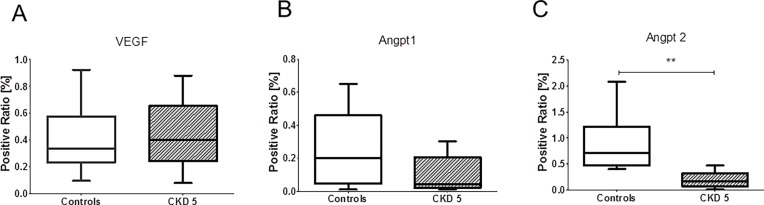
Immunohistochemistry staining. Representative stains for VEGF (**A**), Angpt1 (**B**) and Angpt2 (**C**) in omental biopsies of children with CKD 5 and controls (**B,D,F**)

**Fig 5:**
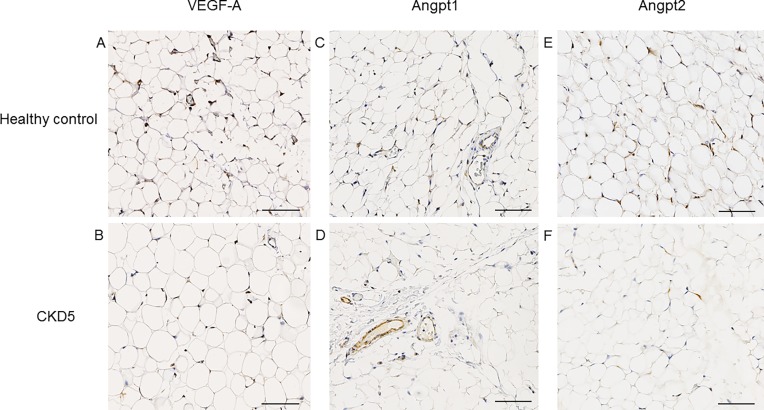
VEGF, Angpt1 and Angpt2 in omental tissue. Quantitative immunohistochemistry for VEGF (**A**), Angpt1 (**B**) and Angpt2 (**C**) in CKD 5 patients and controls. The Angpt2 expression was significantly lower in CKD children (p = 0.0137), Mann-Whitney test.

Hematoxylin-eosin and acid fuchsin orange G staining did not show any signs of inflammation. There was no evidence for autophagy or apoptosis in omental biopsies of CKD patients (Fig 3). To investigate mechanisms involved in the reduction of microvascular density, we analyzed VEGF-A and its receptor, VEGF-R2 in omental biopsies by quantitative immunohistochemical staining. There were no significant differences in controls and CKD patients by staining for VEGF-A (0.41% vs. 0.44%; p = 0.70) or the intracellular domain (0.43% vs. 0.67%; p = 0.61) or extracellular domain (0.43% vs. 0.3%; p = 0.78) of the VEGF receptor 2 (Flk-1/KDR) when examined with specific antibodies against either domain (Figs 4 and 5). Staining for Angpt1 was not significantly different (0.26% vs. 0.11%; p = 0.11), but the Angpt2 expression was significantly lower in CKD children (0.91% vs. 0.21%; p = 0.0137) (Figs 4 and 5), resulting in a ratio of Angpt1/Angpt2 of 0.85 ± 1.2 in CKD and of 0.25 ± 0.25 in controls (p = 0.19). There was no significant correlation of the expression of VEGF-A and Angpt1, Angpt2, or the Angpt1/Angpt2 ratio in patients or controls (data not shown).

## Discussion

There is accumulating evidence that the earliest manifestations of CVD occur at the level of the microcirculation. The microvasculature, a network consisting of a continuum of small resistance arteries, arterioles, capillaries, and venules with a diameter less than ∼200 μm, covers a vast surface area and is actively involved in vital functions of the cardiovascular system, including regulation of perfusion, fluid and solute exchange, hemostasis and coagulation, inflammatory responses, vasculogenesis and angiogenesis [23]. Deficits in microvascular structure or function ultimately result in deficits in tissue perfusion and target organ damage [24]. In patients with CKD, the presence of endothelial dysfunction has been documented consistently [3], but biopsy-based morphological studies of the microcirculation have not been performed. Here we show, by two different methods performed by independent observers, a reduction of microvascular density by 36–51% (depending on the method) in ex-vivo biopsies of the omentum in children with stage 5 CKD prior to dialysis, indicating large-scale rarefaction of the microcirculatory network.

Microvascular density has not been studied previously in omental tissue in CKD patients. It is well known that microvascular rarefaction accompanies arterial hypertension and mediates target organ damage [25]. However, our pediatric patients were not hypertensive and had no classical risk factors for atherosclerosis beyond CKD5 or other age-related comorbidities that might impact the microcirculation. The rarefaction of microvessels of children of all age groups in the omentum, an adipose tissue, in the absence of hypertension could indicate a systemic CKD-induced pathogenesis, and this contention is supported by the finding of microvascular rarefaction in rats with experimental uremia [7, 8, 26] and in the skin [7, 10] and retina [14–17] of patients with CKD. In this perspective, reduced capillary density in CKD patients demonstrated post mortem in the myocardium [11], might be part of a systemic “uremic” microangiopathy and might help explain the extremely high mortality from CVD [27]. Assuming that circulating uremic toxins provide a chronic deleterious environment for endothelial cells of the entire vasculature [28], it is conceivable that microvascular rarefaction occurs early in patients with CKD, preceding the macrovascular pathology. The rarefaction of microvessels as demonstrated in the present study was extensive, whereas overt atherosclerotic changes are generally absent in this age group. Indeed, no pathognomonic changes were seen in biopsies of small arteries obtained from pediatric pre-dialysis CKD patients [29].

Microvascular density assessed by the manual method was inversely associated with age, height and BSA in patients and controls, indicating a major influence of human development on capillary supply of the omentum. This confirms data of a previous study, where we have quantified omental microvessel density by fully automated imaging in normal peritoneum in a larger healthy population (n = >100, age 0.1–60 years) and found a U-shaped curve with highest values in infants below one year of age and in older adults [20]. Interestingly, the BMI-SDS was not associated with microvessel density in either study. Since the BMI is closely related to adipose visceral fat mass in healthy adults [30] and children [31], these data suggest that microvessel density in the omentum of healthy children and the decreased density observed in children with CKD, respectively, are independent of visceral adipose tissue volume in children. This was also confirmed in the multivariate analysis, where age and the serum creatinine level were the only significant independent predictors of microvascular density. While similar associations with age, height and BSA were found in our previous larger study in a healthy population using automated imaging, no statistically significant correlations (except for age) were found for the results of automated imaging in the present study, most likely due to the limited number of samples studied with this method.

Structural remodeling of the microcirculation is a physiological process in response to circulatory demands [32]. Extensive capillary rarefaction as seen in the present study could be due to enhanced endothelial apoptosis as has been described in hypertensive rats in association with oxidative stress [33]. Moreover, reactive oxygen species and/or chronic hyperphosphatemia in CKD could promote apoptosis and also, autophagy [34, 35]. While we could not find evidence for these mechanisms by immunohistochemistry, their role in CKD-induced microvascular rarefaction cannot be ruled out, since the temporary footprints of these processes (caspase-3, LC-3) may be missed [36, 37]. However, physiologic regression of microvessels may occur due to various other mechanisms [38].

Previous studies in CKD patients have shown that CKD is a state of endothelial dysfunction and impaired angiogenesis [39]. Animal models of CKD likewise consistently show impaired angiogenesis. Thus, capillary rarefaction and tissue hypoxia due to progressive experimental glomerular injury [40], in the remnant kidney model [41], in rats with streptozotozin-induced diabetes [42] or in the model of hindlimb ischemia [43] fail to induce an angiogenic response in animals with CKD, i.e. upregulation of vascular endothelial growth factor A (VEGF-A) and its receptor VEGF-R, whereas administration of VEGF-A resulted in improved renal function and lower mortality rates in experimental animals [41]. Moreover, increased serum levels of the soluble VEGF receptor 1 (sFlt-1), a VEGF antagonist, associate with endothelial dysfunction in CKD patients [44].

We could not find a significant difference in VEGF-A in omental capillaries of children with CKD and controls. Likewise, there was no difference in abundance of the main VEGF receptor, VEGF-R2, or of the internal or external part of the receptor. Previous studies in spontaneously hypertensive rats could demonstrate a decrease of the external domain of the VEGF-R2 due to cleavage of VEGF-R2 by metalloproteinases, which are upregulated in CKD [45]. However, our findings do not support a diminished angiogenesis due to VEGF-R2 deficiency. Angiogenesis and endothelial homeostasis are also controlled by the angiopoietins, i.e. Angpt1 and Angpt2. We found no difference in Angpt1 concentrations in omental tissue, but a marked reduction in Angpt2. This finding was unexpected since an increase of the level of circulating Angpt2 has been described in patients with CKD [46–48]. In children, lower serum Angpt1 levels were found in CKD 4–5 and high Angpt2 serum levels on dialysis, resulting in higher serum Angpt2/Angpt1 ratios in children on dialysis but not in pre-dialysis children. In our cohort of children with CKD stage 5 (at start of PD), tissue Angpt2 was 77% lower, and the Angpt1/Angt2 ratio was 3.4 fold higher, albeit not reaching statistical significance due to the high interindividual variations [48]. Thus, serum Angpt1/2 ratios in children with CKD stage 4–5 show the opposite tendency compared to our findings in omental tissue, but both trends did not reach statistical significance. The various angiogenic proteins have complex interactions, and Angpt2 may function as a context-dependent agonist or antagonist in promoting angiogenesis [49], depending on interaction with VEGF [50]. Conceptually, a deficiency of Angpt2 as found in the present study could contribute to a diminished angiogenesis and maladaptation to tissue hypoxia. Further studies are needed to clarify the relationship between circulating angiogenic proteins and their levels in tissues and to identify molecular mechanisms involved in dysfunctional angiogenesis in CKD.

The small number of available biopsies is a limitation of our study. Since an effect of age and body dimensions on microvascular density had to be assumed (and was later confirmed by our results), stored biopsies from CKD patients had to be carefully matched to controls for these criteria, but the number of available biopsies was limited. In addition, data on perinatal parameters (birth weight, gestational age), which may affect microvascular development at later age [51, 52] was not available. Since children with CKD frequently are born premature or small for gestational age [53], we cannot rule out that birth history may be a factor explaining microvascular rarefaction in the CKD group. However, a role of hypertension in mediating these changes seems unlikely, since microvascular density was reduced even more (by 52%) in the CKD group after exclusion of 4 patients treated with antihypertensives. It is a further limitation of the study that the duration of uremia was unknown in many CKD patients and that systemic factors regulating angiogenesis and markers of inflammation could not be measured. Further studies in larger populations are needed to clarify the role of the uremic milieu in promoting microvascular rarefaction. Finally, while intra- and interobserver variability of the manual method were acceptable, there was lesser agreement in the manual and automated measurements, especially at higher counts of microvessels, most likely due to technical differences. However, microvascular density was found significantly reduced in the CKD group with both methods.

In conclusion, we found a profound reduction in microvascular density in the omentum of children with stage 5 CKD associated with diminished Angpt2 signaling. The mean microvascular density was significantly reduced in children with CKD, as measured by independent observers using different methodology. Microvascular rarefaction could be a contributor to endothelial dysfunction and an early manifestation of CKD-induced systemic cardiovascular disease.

## Supporting Information

S1 Fig Automated imaging, overview. (A) Example image of a processed tissue containing several negative and positive nuclei (magnification 20x) within a ROI (green dotted line). (B) The same image tile after image cell segmentation and classification. The overlaid labels represent the results of this step, the different staining categories (green = negative and. red = positive).
(TIF)

S2 Fig Automated imaging, cell classification. High magnification image of the cell classification: (A) Small image part in 40x magnification. (B) The same region after cell segmentation and classification highlighting the detected cells in their corresponding staining class.
(TIF)

S1 Table Diagnoses of the control group (n = 32). (DOCX)

S2 Table Abdominal operations (control group). (DOCX)

S3 Table Renal Diagnoses of CKD patients. (DOCX)

S1 Text Ethics Committee approval and Automated Imaging and Image Processing protocol. (DOCX)
