# Supplementary figures and images for: Reduced Microvascular Density in Omental Biopsies of Children with Chronic Kidney Disease
Source: PLoS One. 2016 Nov 15;11(11):e0166050. doi: 10.1371/journal.pone.0166050 (PMC5113061; doi:10.1371/journal.pone.0166050)

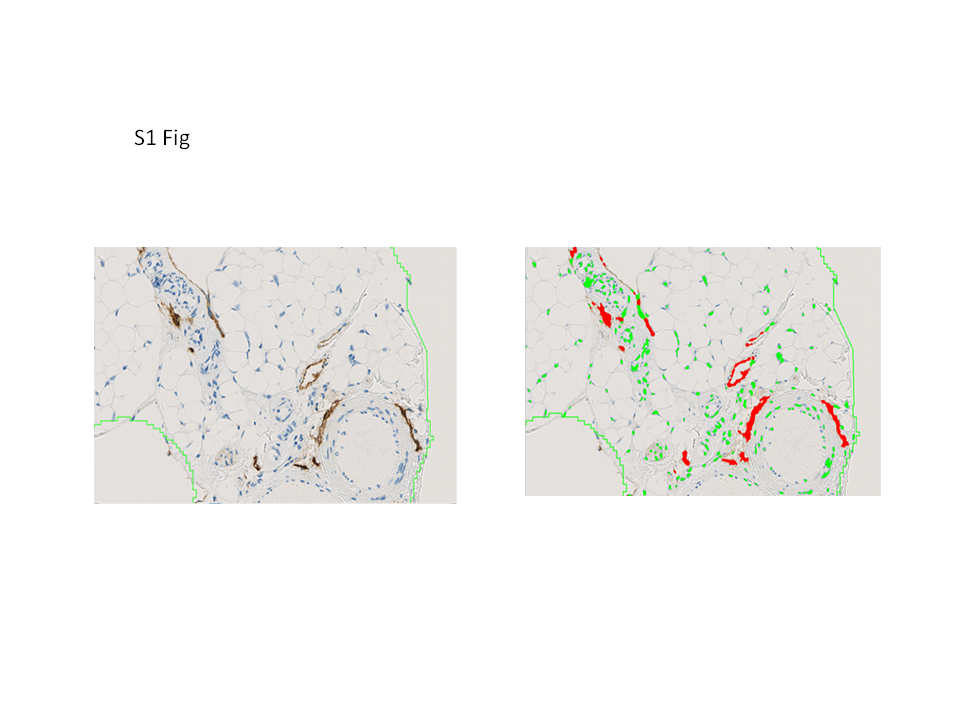

Supplement: S1 Fig — (A) Example image of a processed tissue containing several negative and positive nuclei (magnification 20x) within a ROI (green dotted line). (B) The same image tile after image cell segmentation and classification. The overlaid labels represent the results of this step, the different staining categories (green = negative and. red = positive). (TIF) [file pone.0166050.s001.tif]

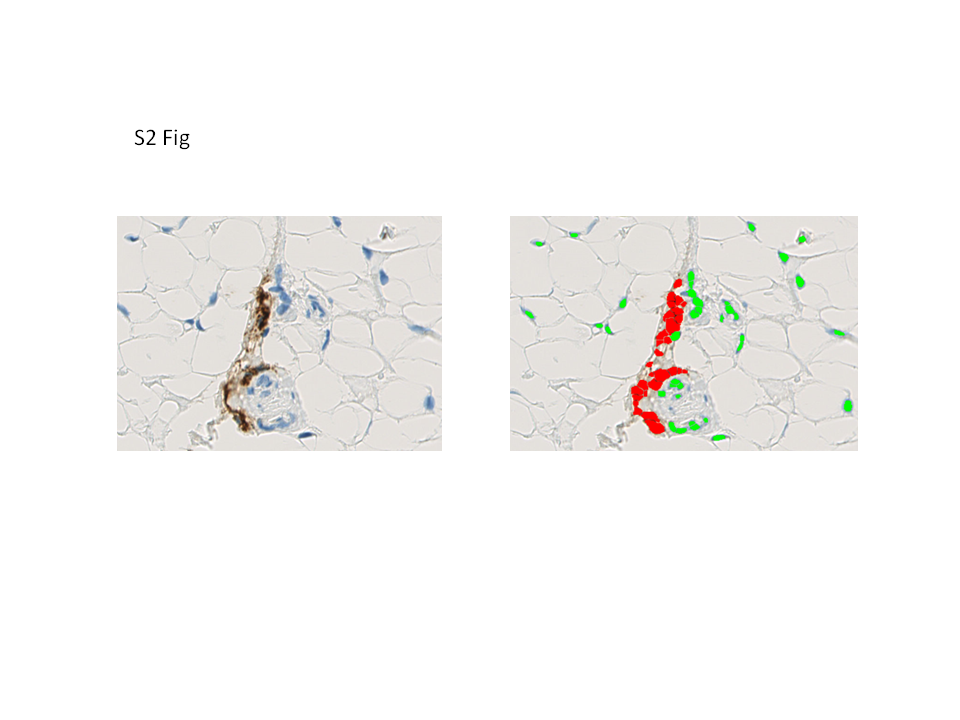

Supplement: S2 Fig — High magnification image of the cell classification: (A) Small image part in 40x magnification. (B) The same region after cell segmentation and classification highlighting the detected cells in their corresponding staining class. (TIF) [file pone.0166050.s002.tif]
