# Supplementary material for: Reduced Microvascular Density in Omental Biopsies of Children with Chronic Kidney Disease
Source: PLoS One. 2016 Nov 15;11(11):e0166050. doi: 10.1371/journal.pone.0166050 (PMC5113061; doi:10.1371/journal.pone.0166050)
Supplement: S1 Table — (DOCX) [file pone.0166050.s003.docx]

**S1 Table. Diagnoses of the control group (n=32)**

| Diagnoses | Total (n) | Percentage (%) |
| --- | --- | --- |
| **Gastrointestinal disorders** | **11** | **34.4** |
| - Gastrooesophageal reflux - Pyloric stenosis - Cholecystolithiasis | 8  1  2 | 72.8  9.1  18.2 |
| **Congenital malformations** | **18** | **56.3** |
| - Anorectal malformations   - Hirschsprung’s disease   - complex malformations - Esophageal atresia - Bladder exstrophy | 13  3  10  1  4 | 72.2  23.1  76.9  5.6  22.2 |
| **Other** | **3** | **9.4** |
| - **Turner‘s Syndrome** - **Sickle cell anemia** | **1**  **2** | **33.3**  **66.7** |
