# Supplementary material for: Reduced Microvascular Density in Omental Biopsies of Children with Chronic Kidney Disease
Source: PLoS One. 2016 Nov 15;11(11):e0166050. doi: 10.1371/journal.pone.0166050 (PMC5113061; doi:10.1371/journal.pone.0166050)
Supplement: S2 Table — (DOCX) [file pone.0166050.s004.docx]

**S2. Table. Abdominal operations (control group)**

|  | *Total (n)* | *Percentage (%)* |
| --- | --- | --- |
| **Upper gastrointestinal tract** | 12 | 37.5 |
| - Fundoplication - Esophageal reconstruction - Pyloromyotomy - Cholecystectomy | 8  1  1  2 | 66.7  8.3  8.3  16.7 |
| Lower gastrointestinal tract | 9 | 28.1 |
| - Enterostomy placement - Revision of enterostomy - Colectomy/Hemicolectomy - Laparoscopy | 1  4  3  1 | 11.1  44.4  33.3  11.1 |
| **Urinary tract** | 7 | 21.9 |
| - Bladder neck reconstruction   - With vesicostomy or   uretercystoneostomy   - Bladder augmentation   - With vesicostomy or   uretercystoneostomy | 2  1  5  3 | 28.6  71.4 |
| **Other** | 4 | 12.5 |
| - Splenectomy | 2 | 50 |
| - **Secondary wound closure** - **Diagnostic laparoscopy** | **1**  **1** | **25**  **25** |
