# Supplementary material for: Reduced Microvascular Density in Omental Biopsies of Children with Chronic Kidney Disease
Source: PLoS One. 2016 Nov 15;11(11):e0166050. doi: 10.1371/journal.pone.0166050 (PMC5113061; doi:10.1371/journal.pone.0166050)
Supplement: S3 Table — (DOCX) [file pone.0166050.s005.docx]

**S3 Table. Renal Diagnoses of CKD patients**

| Diagnoses | *Total (n)* | *Percentage (%)* |
| --- | --- | --- |
| **Hereditary diseases** | **20** | **87** |
| - Obstructive Uropathy   - Urethral valves - Hypoplasia/Dysplasia - Syndromes with kidney involvement   - Bardet-Biedl-syndrome   - Joubert-syndrome - Multicystic-dysplastic kidney - Methylmalonic aciduria - Congenital nephrotic syndrome | 8  6  6  2  1  1  2  1  1 | 40  75  30  10  50  50  10  5  5 |
| **Acquired diseases** | 3 | 13 |
| - Glomerulonephritis   - MPGN Type 2   - IgA-Nephropathy - Tubulopathy | 2  1  1  1 | 66.7  50  50  33.3 |
