# Supplementary material for: Reduced Microvascular Density in Omental Biopsies of Children with Chronic Kidney Disease
Source: PLoS One. 2016 Nov 15;11(11):e0166050. doi: 10.1371/journal.pone.0166050 (PMC5113061; doi:10.1371/journal.pone.0166050)
Supplement: S1 Text — (DOCX) [file pone.0166050.s006.docx]

**Supporting Information**

**Ethics Committee Approval**

Heidelberg is the leading center of the International Pediatric Peritoneal Biopsy Study. Based on the IRB vote obtained from the Ethical Committee of the Medical Faculty Heidelberg, local IRB votes have been obtained in the participating centers (Barcelona, Berlin, Budapest, Lyon, Prague, Vilnius).

**Automated Imaging and Image Processing**

Histological sections were automatically imaged in a 20x magnification (resolution: 0.46 μm/pixel) using the Hamamatsu NanoZoomer 2.0-HT Scan System (Hamamatsu Photonics. Hamamatsu Japan). For the scanning of the glass slides, the slide scanner automatically detects the region of interest that contains the tissue and also determines automatically a valid focal plane for scanning. The resulting digital images (virtual slides) had a file size of up to 25 GB depending on the scanned area,the number of scanned layers and the used magnification. Because of lossless JPEG compressing algorithms, the file size of the virtual slides is reduced enormously (for example. a 20 GB Slide is reduced to 500 MB). The image processing algorithms have been developed using VisiomorphDP version 4.5.1.324 (Visiopharm, Hoersholm, Denmark).

Image processing was performed in several distinguished steps:

(i) ROI detection: Haematoxylin color channel was used to determine regions of interest (ROIs). The haematoxylin color channel was extracted from the RGB images using color deconvolution [1]. A subsesquent mean filter with 3x3 kernel size was used to smooth the resulting images. Automatic thresholding methods [2] were then used to determine the ROIs. To remove small holes within the ROIs morphological closing was applied [3]. As a post-processing step, areas that were too small (small tissue fragments. staining artifacts) were also removed by using morphological operations like opening and closing [3]. A final quality check was done manually by excluding large vessels which are not suitable for subsequent analysis. For VEGF and podoplanin quantification, mesothelium was excluded to prevent false positivity.

(ii) Cell\Staining detection and classification: The first step of the detection involves a segmentation of all nuclei in the previously detected ROIs. Cell nuclei segmentation was done by a watershed segmentation [4,5,] on the haematoxylin colorband provided by color deconvolution algorithm [1]. Detected nuclei were subdivided into negative and positive by simple thresholding on the DAB colorband. As post-processing step, nuclei that were too small where removed by an area filter.

(iii) Cell/Area counting: the output variables from this image processing steps were the number and area of the positive and negative nuclei and the total tissue area

**Aperio Analysis**

Automated quantitative analyses were performed using the Aperio Image Analysis Software (Aperio® Technologies, Inc., Vista, California, USA) and viewed by Image Scope version 11 (v11.2.0.780). Immunohistochemical stainings were evaluated using the Aperio Positive Pixel Count Algorithm (version 9) for quantification of the amount of positive pixels per scanned virtual slide. Input parameters of the algorithm are specific color ranges in the HSI colorspace (Hue value 0.1; Hue width 0.5) and intensity ranges to differentiate between negative, weak, medium and strong staining intensities. Intensity thresholds were optimized and validated specifically for each staining. Positivity was calculated as Total number of positive pixels divided by total number of pixels: (NTotal – Nn) / (NTotal).

**References**

1. Ruifrok A, Johnston D (2001) Quantification of histochemical staining by color deconvolution. Anal. Quant. Cytol. Histol. 23: 291–299.

2. Wang C-W (2011) Robust Automated Tumour Segmentation on Histological and Immunohistochemical Tissue Images. PLoS One 6: 1–8. Available: http://www.pubmedcentral.nih.gov/articlerender.fcgi?artid=3046129&tool=pmcentrez&rendertype=abstract. Accessed 17 Mar 2011.

3. Gonzales RC, Woods RE, Eddins SL (2009) Digital Image Processing using Matlab. Knoxville, Tennessee: Gatesmark Publishing. pp. 209–229, 720–712.

4. Jung C, Kim C (2010) Segmenting clustered nuclei using H-minima transform-based marker extraction and contour parameterization. IEEE Trans. Biomed. Eng. 57: 2600–4. Available: http://www.ncbi.nlm.nih.gov/pubmed/20656653.

5. Beucher S (1992) The watershed transformation applied to image segmentation. Scanning Microsc. Int. 6: 299–314.
